# Prevalence and Predictors of Metabolic Syndrome among Patients with Bronchial Asthma: A Cross Sectional Study

**DOI:** 10.2174/1874306402115010014

**Published:** 2021-06-18

**Authors:** Abdellah H.K. Ali

**Affiliations:** 1 Department of Respiratory Medicine, Sohag Faculty of Medicine, Sohag University, Sohag, Egypt

## Abstract

**Background:**

Recent studies have reported the epidemiological link between Metabolic Syndrome (MS) and asthma, but it has rarely been studied in Egypt. The study aimed to investigate the prevalence of MS and its predictors among asthma patients in Egypt.

**Methods:**

In total, 320 patients with bronchial asthma were included. The following were assessed: spirometric evaluation, anthropometric indices, blood pressure, fasting blood sugar and serum lipid profile. We analyzed the correlation between metabolic scores and patient characteristics. Predictors of MS were identified using logistic regression analysis.

**Results:**

The prevalence of MS was 57.5% in asthma patients. For asthma patients, low High-Density Lipoprotein (HDL) and abdominal obesity were the commonest metabolic abnormality. Waist circumference, Fasting Blood Sugar (FBS) and triglyceride correlated significantly with asthma (P ‹ 0.05). FBS and DBP were the best predictors of MS.

**Conclusion:**

MS is frequent in asthma patients in Egypt. Obesity and lipid abnormalities were the commonest metabolic abnormality. Screening of these patients for components of metabolic syndrome should be a part of routine workup.

## 1 INTRODUCTION

Bronchial asthma and metabolic syndrome are major health problems that have increased rapidly worldwide [1]. Reversible airway obstruction with hyper-responsiveness is the main criteria of bronchial asthma, while essential hypertension, obesity, dyslipidemia, and type-2 diabetes mellitus are the main criteria of MS [2-4].

A quarter of the world’s population is estimated to have MS and increased cardiovascular risk compared with people without it [5]. Diabetes mellitus is 5-fold greater in people with MS [6]. It was found that hyperglycemia and hyper-insulinaemia have clinically relevant negative effects on airway structure, function; subjects exercise capacity and health status [7-9].

To date, the prevalence of metabolic syndrome in asthma subjects has not been studied before in Upper Egypt. Therefore, we aimed to investigate the prevalence and predictors of metabolic syndrome in asthma patients.

## 2 MATERIALS AND METHODS

### 2.1 Study Design

A cross-sectional study on 320 asthma patients was performed. The patients admitted to the Department of Pulmonology, Sohag University Hospital were selected. The protocol of the study was reviewed and approved by the Ethical Committee, Sohag university hospital.

### 2.2 Procedures

Clinical and laboratory findings including history taking, clinical examination, spirometry, blood picture, ESR, liver and kidney functions, lipid profile (HDL-cholesterol, triglycerides), and CRP level were analyzed. Bodyweight, height, BMI (wt in kg/ht in meter^2^), and waist circumference were measured in all patients [10].

### 2.3 Evaluation of MS

The definition of Diabetes Federation (IDF) of metabolic syndrome was used to diagnose MS [11]. MS was diagnosed when at least three of the following criteria were present: 1) waist circumference ≥ 94 cm or ≥ 80 cm; 2) systolic blood pressure ≥ 130 mmHg and/or diastolic blood pressure ≥ 85 mmHg or ongoing therapy for hypertension; 3) fasting glucose > 100 mg/dl or ongoing therapy for elevated glucose levels; 4) HDL < 40 mg/dl in men or < 50 mg/dl in women or specific treatment for this lipid abnormality; 5) triglycerides ≥150 mg/ dl or specific treatment for this lipid abnormality.

### 2.4 Statistical Analysis

Data was collected and analyzed using SPSS v.17.0 (SPSS Inc, Chicago, IL, USA). The ANOVA test was used to assess the differences between the groups; Spearman’s correlation coefficient was used to assess the strength of correlations between variables. To identify independent predictors of MS presence, multivariate logistic regression analysis was conducted. A P value of < 0.05 was considered significant.

## 3 RESULTS

### 3.1 Frequency of MS and its Components

All the biochemical and anthropometric parameters pertaining to the diagnosis of MS are depicted in Table **1**. Using IDF definition, MS was diagnosed in 184 (57.5%) patients with asthma.

**Fig. (1):**
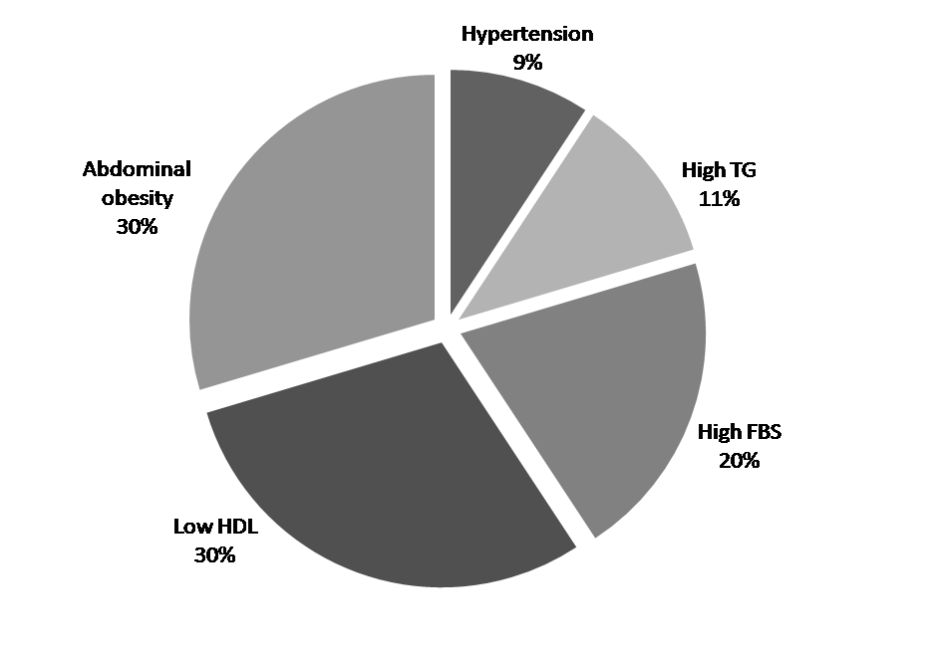
Distribution of the components of metabolic syndrome among the study population FBS:Fasting blood sugar; TG: Triglycerides; HDL: High Density Lipoprotein.

The percent of each component of the MS was estimated in asthma patients. Abdominal obesity was represented in 40%, low HDL-C levels in 40%, elevated fasting glucose levels in 27.5%, elevated triglyceride levels in 15%, and raised blood pressure in 12.50%. So, in asthma patients, the highest percentage was low HDL and abdominal obesity, then FBS (40%, 40%, and 27.5%) respectively, but TG and hypertension were of the lowest incidence (15%, 12.5%, respectively). The most prevalent component in participants with asthma was waist circumference (40%) and Low HDL (40%) (Fig. **1**).

**Fig. (2):**
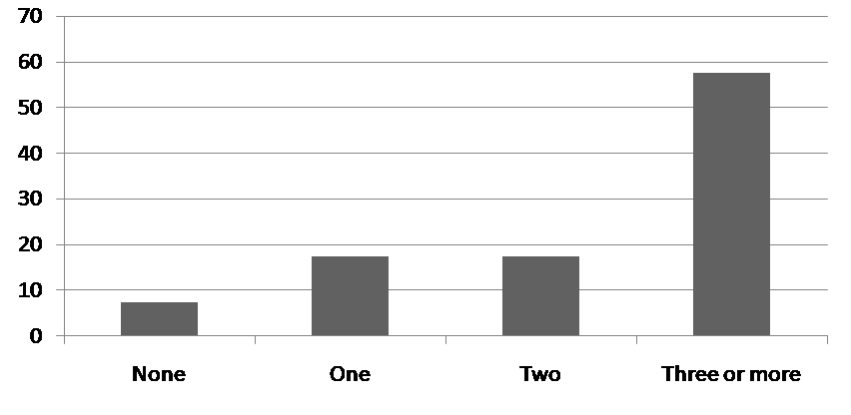
Number of MS componants **MS**; metabolic syndrome.

Asthma patients who did not meet the criteria for MS (n-136), 24 (7.5%) patients had no features of metabolic syndrome while 56 (17.5%) had at least one parameter of metabolic syndrome and 56 (17.5%) had 2 parameters of metabolic syndrome (Fig. **2**).

### 3.2 **Characteristics of Patients with and without** MS

Comparing asthma group who did not have MS and asthma group with MS, there were significant differences between the 2 groups regarding waist circumference (P value= 0.0158), CRP (P value =0.025), DBP (P value =0.0157), triglycerides (P value = 0.0264), and fasting blood glucose (P value <0.0001) (Table **2**).

### 3.3 Correlation between MS and Clinical Parameters

The correlations between clinical parameters and MS observed in subjects affected by MS are shown in Table **3**. In a patient of asthma, FEV1(r = 0.33, P < 0.03), SBP(r = 0.32, P < 0.04), DBP (r = 0.62, P < 0.001) and FBS (r = 0.68, P < 0.001) were positively associated with MS.

### 3.4 Predictors of Metabolic Syndrome

In order to predict the presence of a MS in patients with asthma, logistic regression analysis was done to identify predictors of MS. The independent variables which predict the presence of MS are presented in Tables **3** and **4**. The best predictors of MS in asthma were FBS (P=0.002, OR=0.63, 95% CI: 2.69-5.01), and DBP (P=0.017, OR=0.78, 95% CI: 3.40-5.33) (Table **4**).

## 4 DISCUSSION

Ours is the first study in Upper Egypt to evaluate the prevalence of MS in patients with bronchial asthma and to examine the correlations between MS with comorbidities and asthma characteristics.

The main findings of the current study were (57.5%) patients with asthma had MS. The prevalence of metabolic syndrome varies worldwide; its prevalence is not uniform. It has been reported to be 18–46% in different local studies [12]. 0ur finding was higher than the results obtained from a Nigerian report, where 27% of the participants were found to be victims of MS [13]. Uzunlulu *et al*., studied the prevalence of metabolic syndrome in asthma patients which was 36.7%, being lower than our study [14]. Diverse criteria used to diagnose it and differences between the populations in different studies (physical activity, diet, lifestyle, age, smoking etc**.)** may partly explain the differences in the prevalence of metabolic syndrome.

In our study, the most prevalent component in participants with asthma was waist circumference (40%) and low HDL (40%). A previous study reported that type 2 diabetes mellitus, dyslipidaemia and hypertension, tend to occur more in asthma patients. The prevalence of obesity in asthma patients and the use of steroid therapy by asthmatics may be the cause. Adeyeye *et al*., found that the prevalence of obesity in asthma patients was 49.4% which is higher than our own study [13].

Obesity and asthma are both characterized by inflammation. Obesity leads to a proinflammatory milieu characterized by increased levels of TNF-α, plasminogen activation inhibitor-1, angio-tensinogen, plasminogen activation inhibitor-1, IL-6, leptin, and decreased adiponectin levels which enhance endothelial functions. The TNF-α is also included in the initiation of allergic airway responses in asthma. Meta-analysis has shown the association between obesity and the development of asthma [15]. Asthma is a complex syndrome that encompasses multiple phenotypes. The relationship with obesity has been addressed in the past; however, the underlying mechanism of such a relationship seems to be more complex, and not explained by the body weight alone. The metabolic syndrome carries a condition of systemic inflammation that could potentially explain the influence on asthma onset and severity. This is a rather unexplored area that could potentially open new scenarios in the diagnostic algorithm and in the strategic approach, with a more comprehensive assessment of the disease.

We found a statistically significant difference between patients with MS and patients without MS regarding metabolic parameters but no statistical significance regarding functional parameters. So, the incidence of MS is not related to the severity of the disease. In our study, we did not find any significant association between the presence of MS in asthmatic patients and pulmonary function tests. In a similar study by Adeyeye *et* *al*., it was reported that the pulmonary function test was not affected by the presence of MS. This because that our asthma patients were already diagnosed and were on treatment for asthma [13]. In contrast, Leone found an independent relationship between lung function and MS in both sexes [16].

An interesting observation was that patients with MS in asthma had significantly higher CRP compared with those without MS. Park *et al*., showed that the MS -asthma association was substantially mediated by insulin resistance and systemic inflammation. MS and each MS component were significantly associated with insulin resistance and systemic inflammation [17]. This observation indicates that the presence of MS in asthma patients is associated with more intensive systemic inflammation. In recent studies, it has been reported that a decline in pulmonary function has been linked to increased inflammatory markers [18].

Another objective of the study was to explore the association of individual components of MS with asthma patients. The association with individual components was further explored using the logistic regression models. In a patient of asthma, the data showed a significant positive association with FEV1, SBP, DBP and FBS. These findings suggest that MS-related factors could contribute to the relationship between MS, and asthma. MS components such as abdominal obesity, hyperglycemia [19], dyslipidemia [20] and hypertension [21] accelerate lung function decline. Several studies showed that persons with impaired lung function are at greater risk of developing hypertension, and cardiovascular disease, insulin resistance and diabetes mellitus, within 10 years [22-23]

The last objective of the study was to explore the determinants of association between the individual components of MS and asthma. Among the components of MS; FBS, DBP is the best predictor of MS.

## CONCLUSION

The prevalence of metabolic syndrome is high in patients with BA. It shows the importance of the development of comprehensive strategies aimed at the prevention and treatment of co-morbidities. Correction of the risk factors of MS may have a significant role in the prevention of complications and improvement of quality of life of patients with BA.

## Figures and Tables

**Table 1:** The demographic and metabolic characteristics of the study groups.

**Clinical Parameters**	**Asthma (320)**
Age	45.60 ± 11.11
Sex	-
Females	224 (70%)
Males	96 (30%)
BMI (kg/m2)	23.54 ± 3.18
Waist circumference	97.98 ± 15.55
SBP	133 ± 18.76
DBP	81 ± 10.57
FBS (mg/dl)	151.05 ± 65.36
Triglyceride (mg/dl)	121.41 ± 106.59
HDL-Chol (mg/dl)	49.0 ± 15.8
CRP	6.3 ± 2.1
FEV1	44.85 ± 17.89
FEV1/Fvc	65.15 ± 13.02
Met syndrome	184 (57.5%)

Data are presented as mean ± SD or as frequency and percentage; BMI: Body Mass Index; CRP: C reactive protein; FBS: Fasting blood sugar; SBP: systolic blood pressure; DBP: diastolic blood pressure; LDL: Low Density Lipoprotein; HDL: High Density Lipoprotein; FEV-1: Forced Expiratory Volume in the first second; Mets: Metabolic syndrome.

**Table 2:** Criteria of metabolic syndrome of the study population.

-	**Asthma**		**P value**
**Clinical Parameters**	**MS**	**No MS**	
Age	46.45	44.75	0.63
Females	37.50%	32.50%	0.313
Males	20%	10%	0.053
BMI	23.99 ± 3.72	21.55 ± 4.33	0.065
SBP	138.5 ± 19.40	127.5 ± 16.8	0.06
DBP	87.5 ± 4.729	74.5 ± 10.87	0.001
FEV1	41.95 ± 20.84	47.75 ± 14.3	0.31
FEV1/FVC	63.9 ± 15.9	64.4 ± 9.56	0.72
FBS	191.80 ± 70.39	110.3 ± 17.81	0.001
Triglyceride (mg/dl)	155.4 ± 140.1	87.4 ± 35.37	0.042
HDL-Chol (mg/dl)	58.5 ± 9.5	51.7 ± 7.5	0.47
CRP	7.2 ± 1.54*	5.6 ± 2.69	0.03

MS: Metabolic syndrome; BMI: Body Mass Index; CRP: C reactive protein; FBS: Fasting blood sugar; SBP: systolic blood pressure; DBP: diastolic blood pressure; LDL: Low Density Lipoprotein; HDL: High Density Lipoprotein; FEV-1: Forced Expiratory Volume in the first second.

**Table 3:** Correlation between clinical parameters and MS.

**Test parameter**	**Asthma with MS**	
	**r**	**P**
Age	0.05	0.74
Sex	0.1	0.5
BMI	0.26	0.1
SBP	0.32	0.04
DBP	0.62	0.001
FEV1	0.33	0.03
FEV1/FVC	0.24	0.13
FBS (mg/dl)	0.68	0.001
Total chol (mg/dl)	0.24	0.13
riglyceride (mg/dl)	0.18	0.26
HDL-Chol (mg/dl)	0.08	0.59
CRP	0.01	0.93

MS: Metabolic syndrome; BMI: Body Mass Index; CRP: C reactive protein; FBS: Fasting blood sugar; SBP: systolic blood pressure; DBP: diastolic blood pressure; LDL: Low Density Lipoprotein; HDL: High Density Lipoprotein; FEV-1: Forced Expiratory Volume in the first second; FVC: Forced vital capacity.

**Table 4:** Outcomes of logistic regression on asthma.

	**OR**	**CI**	**P-value**
**FBS**	0.63	2.69-5.01	**0.002**
**DBP**	0.78	3.40-5.33	**0.017**

DM: Diabetes mellitus; DBP: diastolic blood pressure; FBS; fasting blood sugar; OR; Odds ratio; CI; confidence interval.
